# Neurobiological and Microbiota Alterations After Bariatric Surgery: Implications for Hunger, Appetite, Taste, and Long-Term Metabolic Health

**DOI:** 10.3390/brainsci15040363

**Published:** 2025-03-31

**Authors:** Sebastián Chapela, Ludwig Alvarez-Córdova, Andres Martinuzzi, Rosario Suarez, Victoria Gonzalez, Ezequiel Manrique, Janeth Castaño, Gianluca Rossetti, Luigi Cobellis, Vincenzo Pilone, Evelyn Frias-Toral, Luigi Schiavo

**Affiliations:** 1 Departamento de Bioquímica Humana, Facultad de Medicina, Universidad de Buenos Aires, Ciudad Autónoma de Buenos Aires C1121ABG, Argentina; sebachapela@gmail.com; 2 Unidad de Soporte Nutricional, Hospital Británico de Buenos Aires, Ciudad Autónoma de Buenos Aires C1280AEB, Argentina; 3 Facultad de Ciencias de la Salud, Universidad de las Américas (UDLA), Quito 170513, Ecuador; 4 Unidad de Soporte Nutricional, Sanatorio Rio Negro, Rio Negro R8500BAD, Argentina; andres.martinuzzi@nutrihome.com.ar; 5 Asuntos Profesionales y Educación, Fresenius Kabi Argentina, Ciudad de Buenos Aires C1428AAU, Argentina; 6 School of Medicine, Universidad Técnica Particular de Loja, Calle París, San Cayetano Alto, Loja 110107, Ecuador; rsuarez2@utpl.edu.ec; 7 Unidad de Soporte Metabólico y Nutricional, Sanatorio Allende, Córdoba X5000BFB, Argentina; vgonzalez@sanatorioallende.com; 8 Facultad de Ciencias de la Salud, Universidad Católica de Córdoba, Córdoba X5000IYG, Argentina; 9 Unidad de Soporte Nutricional, Hospital Privado Universitario de Córdoba, Córdoba X5016KEH, Argentina; ezequiel.manrique@hospitalprivado.com.ar; 10 Pediatrics, Family Medicine Department, Indiana University Health, Lafayette, IN 47905, USA; jcastano@iuhealth.org; 11 General and Bariatric Surgery Unit, Abano Terme Policlinic, 35031 Padova, Italy; gianlucarossetti@yahoo.it; 12 Unit of General Surgery, Casa Di Cura “Prof. Dott. Luigi Cobellis”, 84078 Vallo Della Lucania, Italy; luicobellis@yahoo.it; 13 Public Health Department, Naples “Federico II” University, AOU “Federico II”, Via S. Pansini 5, 80131 Naples, Italy; vincenzo.pilone@unina.it; 14 Escuela de Medicina, Universidad Espíritu Santo, Samborondón 0901952, Ecuador; evelynft@gmail.com; 15 Division of Research, Texas State University, 601 University Dr, San Marcos, TX 78666, USA; 16 Department of Medicine, Surgery and Dentistry “Scuola Medica Salernitana”, University of Salerno, 84081 Baronissi, Italy; 17 NBFC—National Biodiversity Future Center, 90133 Palermo, Italy

**Keywords:** bariatric surgery, microbiota, appetite regulation, gut–brain axis, GLP-1, ghrelin, food cravings, hormonal changes, metabolic health, short-chain fatty acid

## Abstract

Bariatric surgery (BS) is an effective intervention for obesity, inducing significant neurobiological and gut microbiota changes that influence hunger, appetite, taste perception, and long-term metabolic health. This narrative review examines these alterations by analyzing recent findings from clinical and preclinical studies, including neuroimaging, microbiome sequencing, and hormonal assessments. BS modulates appetite-regulating hormones, reducing ghrelin while increasing glucagon-like peptide-1 (GLP-1) and peptide tyrosine-tyrosine (PYY), leading to enhanced satiety and decreased caloric intake. Neuroimaging studies reveal structural and functional changes in brain regions involved in reward processing and cognitive control, contributing to reduced cravings and altered food choices. Additionally, BS reshapes the gut microbiota, increasing beneficial species such as *Akkermansia muciniphila*, which influence metabolic pathways through short-chain fatty acid production and bile acid metabolism. These findings highlight the complex interplay between the gut and the brain in post-surgical metabolic regulation. Understanding these mechanisms is essential for optimizing post-operative care, including nutritional strategies and behavioral interventions. Future research should explore how these changes impact long-term outcomes, guiding the development of targeted therapies to enhance the recovery and quality of life for BS patients.

## 1. Introduction

According to the Centers for Disease Control and Prevention (CDC), obesity, defined as a body mass index (BMI) of 30 or more, is one of the most prevalent chronic diseases, currently affecting more than 40% of US adults [1]. This complex disorder, which has multiple contributing factors [2,3], affects various systems and organs in the body. Obesity is linked to a wide range of comorbidities, including nonalcoholic fatty liver disease, cardiovascular diseases, type 2 diabetes mellitus, metabolic syndrome, obstructive sleep apnea (OSA), osteoarthritis, depression, chronic kidney disease, hypertension, hyperlipidemia, and some types of cancer. These conditions are associated with an increased risk of death [1,4,5,6,7,8,9,10,11,12].

Bariatric surgery (BS) is widely recognized as an effective strategy for reducing obesity-related comorbidities [13,14,15,16,17,18,19,20,21]. For example, compared to conventional medical therapy, BS significantly improves and reduces the incidence of type 2 diabetes in individuals with obesity [13,14,22,23,24,25]. Among the different procedures, the Roux-en-Y gastric bypass (RYGB) yields the most notable results, with an average weight reduction of 15–30%, depending on the type of surgery performed [13,14,26,27,28,29,30]. Additionally, BS has been reported to lower the prevalence of metabolic syndrome [13,14,31,32,33,34].

These surgeries not only promote weight loss by reducing the gastric capacity—leading to increased satiety and malabsorption—but also have additional long-term effects [35]. Some of these include alterations in taste perception, which influence dietary intake [36,37,38], changes in food cravings [39,40], and modifications in gut microbiota that affect both nutrient absorption and the gut–brain axis [41,42,43,44].

This review provides a comprehensive and novel analysis of the long-term physiological changes following bariatric surgery (BS), addressing a critical gap in the current literature. Unlike previous reviews that primarily focus on short-term outcomes, this work examines the lasting impact of BS on metabolic health. It explores the brain mechanisms that regulate hunger and appetite, the neural adaptations induced by BS, and the sustained changes in food intake behavior. Additionally, this review uniquely investigates the long-term interplay between BS, gut microbiota composition, appetite regulation, and taste perception, highlighting their collective role in sustained weight management and metabolic homeostasis.

## 2. Materials and Methods

This narrative review examines post-bariatric surgery changes, with a particular focus on microbiota alterations and their association with behavioral and neurological changes. A comprehensive literature search was conducted in the PubMed database using a combination of relevant keywords, including “microbiota”, “bariatric surgery”, “behavior”, and “neurological changes”.

Titles and abstracts of the identified articles were screened for relevance by designated research team members assigned to each section. Articles deemed pertinent were selected for full-text review. Additionally, the reference lists of the identified articles were examined to identify and include any additional publications that met the inclusion criteria.

After identifying all relevant articles, the research team categorized them based on the association between post-bariatric surgery microbiota changes and behavioral or neurological changes in specific conditions.

## 3. Mechanisms of Hunger and Appetite Regulation in the Brain

Before discussing the physiological mechanisms that regulate food intake, a few key concepts must be highlighted [45]. Hunger, the physiological urge to eat, arises during fasting to maintain energy balance [41,42,43,46]. In contrast, hedonic hunger—or appetite—refers to eating for pleasure rather than physiological need. These processes are mediated by distinct mechanisms and regulated differently within the brain [45]. Additionally, a third process, known as microbiota-guided hunger, has been described by several researchers. This form of hunger is driven by bacterial metabolites, such as short-chain fatty acids (SCFAs) and human-like peptides [45,47]. Notably, elevated blood leptin levels have been associated with reduced microbiota diversity in both lean individuals and those with obesity [45]. Furthermore, prebiotics appear to suppress appetite by stimulating the production of GLP-1 and PYY, which in turn inhibit ghrelin secretion [45]. Lastly, postbiotics—metabolites derived from the microbiota, such as SCFAs—may influence pancreatic function by suppressing insulin secretion while stimulating appetite through ghrelin signaling [45].

Feeding behavior is regulated through both direct and indirect mechanisms [48]. Direct control occurs via the release of gastrointestinal (GI) peptides in response to nutrient accumulation during a meal [48]. Indirect satiety mechanisms, on the other hand, are triggered in the brain, reducing food intake through meal-induced feedback [48]. The hindbrain plays a crucial role in integrating descending input from the central nervous system (CNS) with ascending signals from the GI tract to regulate eating behavior [48]. Hunger and satiety are governed by multiple CNS regions and cell types [48], forming a complex network of interconnections and neurotransmitters [49,50]. A key structure in this network is the Arcuate Nucleus (ARC), located in the hypothalamus along the ventral surface surrounding the third ventricle [49]. Within the ARC, two distinct neuronal populations exert opposing effects on feeding behavior: pro-opiomelanocortin (POMC) neurons suppress food intake, while agouti-related peptide (AGRP) neurons induce intense hunger, even in satiated mice [49,51]. Food deprivation strongly activates AGRP neurons through glutamatergic afferents, particularly from the paraventricular hypothalamic nucleus (PVH), a major source of excitatory input [49]. Interestingly, even sensory exposure to food rapidly inhibits AGRP neuron activity [49]. Another critical region in feeding regulation is the lateral hypothalamic area (LHA), located dorsolateral to the ARC. The activation of GABAergic LHA neurons significantly increases food-seeking behavior, particularly via their projections to the ventral tegmental area (VTA), a region implicated in food reward and motivation [49]. Conversely, inhibition of these neurons suppresses feeding urges by extending projections to the lateral habenula [49].

Other key regions involved in appetite regulation are located in the brainstem, including the parabrachial nucleus (PBN) and the nucleus tractus solitarius (NTS). The PBN, situated at the pons–midbrain junction, plays a crucial role in appetite suppression by projecting signals to the amygdala. Notably, AGRP neurons have been shown to inhibit the PBN, influencing feeding behavior [49]. Both the PBN and NTS receive satiety signals from the periphery and help modulate food intake [49]. Multiple neurotransmitters participate in the regulation of these mechanisms. Serotonin (5-HT) is linked to appetite suppression, while norepinephrine modulates the activity of AGRP and POMC neurons [49]. Dopamine also plays a critical role, particularly in food reward, by acting on the ventral tegmental area (VTA) and the nucleus accumbens (NAc) [49,52]. Research suggests that reward-related regions (such as the NAc) and cognitive–emotional centers (including the medial prefrontal cortex and amygdala) interact with homeostatic centers (ARC and LHA) to regulate eating behavior even in the absence of caloric need [48].

Another key factor influencing appetite is ghrelin, an orexigenic peptide composed of 28 amino acids, which stimulates food intake in both humans and rodents [48]. Ghrelin is primarily produced and released by the stomach’s X/A-like oxyntic cells, serving as the main source of circulating ghrelin, which passively diffuses into the central nervous system (CNS) [48]. In both humans and animals, exogenous ghrelin administration increases hunger, body weight, and food consumption [48]. Additionally, ghrelin enhances the intake of palatable foods and promotes food-seeking behavior by acting on the ventral tegmental area (VTA), highlighting its role in both hedonic eating and homeostatic re-feeding [48].

Ghrelin is not the only peripherally secreted hormone that regulates appetite. Other hormones, such as incretin, insulin, cholecystokinin (CCK), and peptide tyrosine-tyrosine (PYY), also provide crucial hormonal feedback on food intake [53]. These hormones play an essential role in the gastrointestinal (GI) process by breaking down food particles into smaller sizes, facilitating gastric storage and digestion before they reach the small intestine [53]. This process typically lasts between 30 and 60 min, meaning that humans often stop eating long before significant amounts of nutrients reach the colon [53]. Consequently, “satiation”—the sensation of fullness that signals the end of a meal—is primarily driven by the stomach, as more distal digestive processes are yet to be activated [53]. PYY, a polypeptide hormone primarily produced in the neuroendocrine L cells of the distal intestine, shares its site of synthesis with glucagon-like peptide-1 (GLP-1). Released postprandially in response to food intake, PYY inhibits upper GI motility and colonic transit, promoting satiety [54]. It also plays a role in central energy regulation by acting on the nucleus of the solitary tract (NST) and the lateral hypothalamus [55], where it reduces the release of neuropeptide Y (NPY), a potent appetite stimulant [56]. GLP-1 is well known for its appetite-suppressing effects. It directly influences neuronal pathways that regulate food intake, enhancing satiety while also slowing gastric motility. By delaying stomach emptying, GLP-1 prolongs the feeling of fullness and further reduces the urge to eat [56].

Adipose tissue is the primary source of leptin, a hormone released into the bloodstream in proportion to the body’s total fat stores [57]. Its secretion is also influenced by factors such as sudden changes in calorie intake. Notably, leptin levels can decrease by 10–20% within just three days of fasting, even before a significant reduction in adipose tissue mass occurs [57]. Leptin exerts its primary effects in the brain and secondary effects in the peripheral nervous system [57]. By binding to and activating the long-form leptin receptor (ObRb), it directly influences regions of the hypothalamus that are not protected by the blood–brain barrier [57]. Preclinical research suggests that leptin primarily stimulates pro-opiomelanocortin (POMC) neurons in the hypothalamic arcuate nucleus (ARC), playing a key role in appetite regulation [57,58].

## 4. Impact of Bariatric Surgery on Brain Responses to Hunger and Appetite

### 4.1. Hormonal Changes After Surgery

Bariatric surgery (BS) leads to significant alterations in gastrointestinal hormones that regulate appetite and metabolism, contributing to reduced hunger and increased satiety [42]. A meta-analysis has shown that BS significantly decreases ghrelin and leptin levels while increasing GLP-1 and PYY levels. However, different surgical methods yield varying outcomes. Sleeve gastrectomy (SG), Roux-en-Y gastric bypass (RYGB), biliopancreatic diversion without duodenal switch (BPD), and gastric banding (GB) significantly reduce leptin levels, whereas one-anastomosis gastric bypass (OAGB) shows no significant effect (standard mean deviation (SMD) = −9.98, 95% confidence interval (CI): −24.90 to 4.94, *p* = 0.19) [59]. Regarding ghrelin levels, the meta-analysis found that SG significantly decreases ghrelin (SMD = −1.35, 95% CI: −2.00 to −0.69, *p* < 0.00001), while BPD increases it (SMD = 0.59, 95% CI: 0.25–0.94, *p* = 0.0007). In contrast, RYGB and GB do not significantly alter ghrelin levels [59]. Other studies also confirm that ghrelin typically decreases after SG, contributing to reduced appetite and food intake [60,61,62]. Conversely, RYGB may not consistently lower ghrelin levels, yet it still results in significant weight loss, suggesting alternative mechanisms at play [63]—which may explain differences in appetite regulation between these two procedures [61].

The reduction in leptin levels after BS is also associated with improved leptin sensitivity, indicating a decrease in the adipose tissue mass and an enhanced ability of the body to regulate hunger and energy expenditure [59,64].

On the other hand, GLP-1 exhibits a significant increase in both fasting and postprandial levels following RYGB and SG [60,61,65]. This rise is associated with improved glucose homeostasis and reduced appetite [66]. Additionally, PYY, a hormone released by distal gut cells, suppresses appetite by slowing gastric emptying and reducing gut motility. Studies have shown that PYY levels increase after BS (SMD = 0.63, 95% CI: 0.20 to 1.06, *p* = 0.004), particularly following RYGB [59].

### 4.2. Brain Imaging Studies Post-Surgery

Brain imaging studies, including functional Magnetic Resonance Imaging (fMRI) and Positron Emission Tomography (PET), have provided valuable insights into the neural changes related to hunger and satiety following BS. These studies primarily examine how BS affects the brain regions involved in reward processing and cognitive control, shaping eating behavior [67,68,69]. In an original investigation, Wang et al. found that after LSG, habenular gray matter volume (Hb GMV) increased to levels comparable to normal-weight controls and was associated with a lower BMI [67]. Additionally, before LSG, patients exhibited higher resting-state functional connectivity (RSFC) between the Hb and the insula, precentral gyrus, and rolandic operculum compared to controls, but lower RSFC with the thalamus, hypothalamus, and caudate [67]. LSG normalized the connection patterns, and a reduced Hb-insula RSFC correlated to decreased BMI, lower food addiction scores, and less emotional eating, while increasing the Hb–thalamic and Hb–hypothalamic RSFC, which were linked to decreased hunger and lower BMI [67].

A systematic review of the fMRI studies has shown that after RYGB, the food cue reactivity in the brain regions associated with reward processing, such as the caudate, putamen, and nucleus accumbens (NAc), either decreases or remains unchanged. This suggests a reduction in the rewarding effects of food, which may contribute to decreased appetite and food intake [69,70]. Additionally, evidence indicates an increased activation in the dorsolateral prefrontal cortex (dlPFC), suggesting enhanced cognitive control over eating behavior [67]. Notably, RYGB produces more significant changes than SG, including greater activation in the dlPFC and reduced activation in the parahippocampal/fusiform gyrus in response to high-energy food cues. This implies stronger cognitive dietary inhibition and decreased attention to calorie-dense foods following RYGB compared to SG [67]. Table 1 presents a comparative analysis of the brain effects produced by RYGB and SG.

Original studies indicate that RYGB leads to a greater reduction in brain reward center activation in response to food cues compared to weight loss achieved through very-low-calorie diets (VLCs) [71]. This reduction is linked to improved engagement of the homeostatic appetite regulation system following RYGB-induced weight loss [71]. Additionally, resting-state fMRI studies suggest that BS, including SG, decreases functional connectivity in brain regions involved in self-referential processing and interoception, such as the ventromedial prefrontal cortex and insula [72]. These changes correlate with lower BMI and enhanced control over eating behaviors [72].

### 4.3. Neuroplasticity in Appetite Regulation

Recent brain imaging studies reveal significant neuroplastic changes in appetite regulation following BS, particularly in regions governing reward processing, cognitive control, and homeostasis [69]. One study found that gastric bypass surgery induces widespread neural plasticity, altering gray matter density in the hypothalamus, lateral orbitofrontal cortex, and somatosensory cortex—areas crucial for homeostatic control, reward, and sensory processing [73]. These structural changes correlate with weight loss, suggesting a reorganization of the brain networks that regulate eating behavior [73,74].

fMRI studies indicate that RYGB selectively reduces neural responses to high-calorie food cues within the mesolimbic reward pathway, including the caudate, putamen, and nucleus accumbens. This attenuation is linked to a decreased desire for high-calorie foods, reflecting altered reward processing [70,75]. Additionally, as previously mentioned, increased activation in the dlPFC suggests enhanced cognitive control over eating behavior, potentially improving appetite regulation post-surgery [67,70].

Another key pathway influencing post-BS neuroplasticity involves dopamine-mediated reward circuits, which are modulated by gut microbiome alterations. Changes in the gut–brain communication—driven by shifts in gut hormones and microbial composition—play a crucial role in neuroplastic adaptations. BS significantly alters the gut microbiome, notably modifying the Firmicutes/Bacteroidetes ratio and increasing beneficial bacteria like *Akkermansia* [54]. Additionally, elevated satiety hormones such as GLP-1 and PYY, alongside gut microbiota shifts, contribute to reduced food cue reactivity in brain reward systems, ultimately shaping appetite and food preferences [55].

These findings underscore the complex interplay between structural and functional brain changes and hormonal regulation in the success of bariatric procedures.

## 5. Alterations in Food Intake Behavior After Surgery

BS alters the GI tract, significantly influencing the GI hormones, bile fluids, and microbiota, which in turn affect eating behavior and food intake [61]. These changes begin with reduced energy intake and calorie consumption, leading to improved dietary adherence and decreased appetite perception over time [76].

### 5.1. Changes in Food Preferences

Post-operative counseling guides BS patients through dietary transitions, initially prescribing a soft, easily digestible diet followed by a reduced-calorie plan. While specific guidelines vary by hospital and country, they generally align with the bariatric nutritional pyramid recommendations [77]. Before and after the surgery, patients transition to a liquid diet to optimize surgical outcomes, as adherence to preoperative dietary guidelines is crucial for successful weight loss [78,79]. Bariatric dietary prescriptions emphasize easily digestible foods and controlled calorie intake, typically following a 109 kcal plan comprising 25% protein, 33% fat, and 42% carbohydrates [77]. Food choices may vary across medical centers and regions but adhere to fundamental BS dietary principles [77].

Procedures such as RYGB and SG significantly influence food choices and eating behaviors, particularly by reducing the consumption of energy-dense and high-fat foods [80]. RYGB has been associated with better dietary quality, as patients tend to avoid high-energy options more effectively than with other procedures [81]. Research indicates that both SG and RYGB lead to increased protein intake and reduced fat consumption in the postoperative period [82]. Additionally, food preferences evolve significantly after BS, with notable changes persisting for up to five years. Interestingly, patients report a decline in the hedonic appeal of foods, suggesting an altered taste perception over time [82]. The SG procedure, in particular, often modifies food preferences by reducing cravings for sweets and high-energy foods. These changes result from physiological adaptations, including alterations in intestinal hormone profiles, leading to a state of reduced appetite [83]. Notably, individuals who experienced preoperative food cravings show a significant decrease in these episodes post-surgery, along with diminished hedonic responses, regardless of weight loss [37].

Despite the observed changes in food preferences and taste following BS, the underlying mechanisms remain unclear. Future research should focus on elucidating the molecular, hormonal, and neural pathways driving these alterations. Additionally, a deeper understanding of gustatory perception and endocrine–metabolic changes could provide valuable insights into the mechanisms influencing dietary preferences post-surgery [84].

### 5.2. Mechanisms Underlying Decreased Cravings and Changes in Reward-Related Eating Behavior

BS has been shown to modify taste preferences in individuals with prior obesity, likely by increasing anorexigenic gut hormone levels, which may enhance gustatory sensitivity [85]. In this context, leptin is considered a key regulator of sweet taste perception. After BS, decreased plasma leptin levels may alter sweetness preferences, potentially reducing sugar intake [86]. Research indicates a significant association between lower serum leptin levels and a reduced threshold for detecting sweetness during weight loss in both healthy individuals and females with obesity [87]. Food craving episodes pose a major challenge for bariatric patients, as they can significantly impact body weight, particularly in individuals with strong craving tendencies, making weight loss more difficult [39]. Studies show that BS can reduce food cravings more effectively than in non-surgical counterparts [40], especially cravings for high-energy foods, such as sweets and fats. This reduction may be attributed to changes in food choices rather than direct gut-related surgical effects, particularly within the first year post-surgery [88]. Additionally, both SG and RYGB demonstrate better long-term improvements in reducing the food craving frequency compared to procedures like LAGB [89].

### 5.3. Psychological Factors Influencing Food Intake

The long-term success of body weight loss after BS is largely influenced by factors such as emotional eating, loss of inhibition, and overall psychological functioning [76]. These findings align with evidence suggesting that early postoperative physiological changes play a crucial role in shaping patients’ food cravings [90]. Individual adaptations following BS are closely linked to psychological eating behavior traits, which can either facilitate or hinder weight loss efforts. These traits can be assessed using validated clinical tools such as the Three-Factor Eating Questionnaire (TFEQ) [91,92] and the Dutch Eating Behavior Questionnaire (DEBQ) [91]. The TFEQ evaluates cognitive restraint, disinhibition, and perceived hunger, while newer versions also assess uncontrolled and emotional eating—both of which have been associated with post-surgical outcomes and weight loss success [92]. Similarly, the DEBQ measures restraint and emotional eating, with high scores in emotional and external eating patterns negatively impacting BS outcomes [91]. Evidence from TFEQ data suggests that procedures like RYGB, LGB, and SG lead to reductions in uncontrolled and emotional eating, as well as decreased hunger levels [92,93]. However, cognitive restraint may increase or remain unchanged, while disinhibition and hunger scores tend to be higher. These elevated scores have been linked to lower protein intake and increased consumption of dietary fat and overall calories [94,95].

Adjustments in insulin resistance, basal glycemic levels, rapid nutrient digestion and absorption, and changes in gut hormone responses influence craving episodes, particularly during the first year post-surgery [90,96]. These physiological mechanisms are closely linked to patient-reported cravings, especially within the first three months following the procedure [97]. However, research on postoperative food choices, taste changes, and food cravings remains limited and is primarily focused on the RYGB procedure [97].

**Figure 1:**
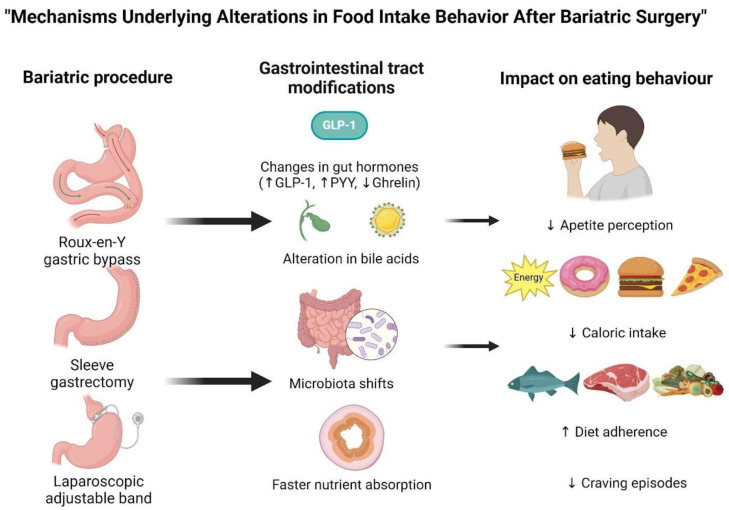
The figure depicts the progression of physiological and behavioral changes that occur after bariatric surgery (BS), including Roux-en-Y gastric bypass (RYGB), sleeve gastrectomy (SG), and laparoscopic adjustable gastric banding (LAGB). These surgical procedures alter the gastrointestinal (GI) tract, triggering remarkable changes in gut hormones (GLP-1 and PYY ↑, ghrelin ↓), bile acid modification, gut microbiota, and the amount of nutrient absorption. These modifications influence eating behavior by lowering appetite perception and energy intake and improving dietary adherence [82,90,100].

Qualitative studies on weight loss trajectories in BS patients highlight the vulnerability to weight regain after the “honeymoon period” of the procedure. Once the initial restriction in food capacity diminishes, patients must actively manage their food intake [98]. Individual variations in eating behaviors and appetite regulation may explain different post-surgical weight loss outcomes [98]. BS significantly improves blood glucose levels within days, independent of weight loss. Compared to VSG and RYGB, LAGB is less effective in reducing hunger and enhancing satiety due to differences in gut hormone responses, making DMT2 remission less frequent. Remission is associated with increased insulin sensitivity in both hepatic and peripheral tissues. Additionally, VSG and RYGB improve insulin secretion and elevate postprandial GLP-1 levels. The vagal pathway may also contribute to appetite and glucose metabolism regulation after BS [99]. Weight loss success depends on patient adherence to nutritional guidelines and effective food intake management [76]. During the first two years post-BS, weight loss is primarily driven by gastrointestinal modifications, leading to reduced energy consumption, improved dietary adherence, appetite suppression, and enhanced psychological well-being [76]. After this period, the risk of weight regain increases as greater cognitive effort is required to regulate food intake [76]. Long-term weight management is influenced by eating habits, psychological health, and overall well-being. Continuous psychological and nutritional support is essential to prevent weight regain and ensure sustained success [76]. Figure 1 summarizes the mechanisms of food intake alterations after BS.

## 6. Effects of Bariatric Surgery on Microbiota and Its Role in Appetite Regulation

### 6.1. Alterations in Gut Microbiota Composition

An experimental and comparative metagenomic study investigating the relationship between gut microbiota and obesity found a 50% lower relative abundance of the phylum Bacteroidetes in mice genetically predisposed to obesity, alongside a proportional increase in Firmicutes. This suggests a microbial composition shift associated with obesity [101,102]. One of the most common changes reported after BS is a relative decrease in Firmicutes and an increase in Bacteroidetes and Proteobacteria [103,104]. According to a prospective longitudinal study in healthy lean subjects and BS patients, these microbial shifts appear to be more pronounced after RYGB compared to other procedures [105]. This is likely due to RYGB causing greater disruption of the GI tract [103]. A comparative observational study investigating the effects of different BS procedures found that Proteobacteria levels increased six months post-RYGB and SG. However, Bacteroidetes abundance increased after RYGB but decreased after SG, suggesting these changes result from the physiological rearrangement of the GI tract [106].

Multiple changes in the gut microbiome have been observed following BS. Dang et al., in a prospective study comparing individuals post-RYGB, post-SG, and controls, reported reduced microbial diversity, with an increase in Proteobacteria and Verrucomicrobiota and a decrease in Firmicutes. However, individuals who underwent SG exhibited limited effects on gut microbiome composition [107]. Additionally, a systematic review of 14 clinical studies (222 participants) by Davies et al. found significant alterations at the phylum, genus, and species levels post-BS. Specifically, SG was associated with a decrease in Firmicutes, whereas RYGB led to an increase in Bacteroidetes and Proteobacteria [108]. Similarly, Gou et al. conducted a systematic review on microbiome modulation after BS, reporting notable microbial shifts, including an increase in Bacteroidetes, Fusobacteria, Verrucomicrobia, and Proteobacteria. Within Firmicutes, specific groups such as Lactobacillales and Enterococcus increased, while others, including Clostridiales, Clostridiaceae, Blautia, and Dorea, showed a decrease [109]. Despite these findings, microbial diversity post-BS remains a topic of debate, as some studies report an increase, while others do not reach a consensus [109]. The key intestinal microbiota changes after BS are summarized in Table 2.

### 6.2. Microbiota’s Influence on Appetite, Food Intake, and Taste Perception

The microbiota–gut–brain axis acts as a bidirectional communication pathway between the gut and the brain, where alterations in gut microbiota can impact various signaling mechanisms [110,111,112]. Vagal afferents located in the intestinal epithelium play a crucial role in this process, as they interact with intestinal bacteria and their metabolites [113]. These vagal pathways facilitate the transfer of information from the gut to neural networks in the nucleus of the solitary tract (NST) and the hypothalamus, which are key regulators of food intake and appetite control [114].

Changes in microbial composition following bariatric surgery (BS) significantly influence gut–brain axis signaling pathways, primarily through vagus nerve-mediated bidirectional communication with the nucleus of the solitary tract (NST) and lateral hypothalamus. These effects are driven by microbiota-derived metabolites and a reduction in peripheral and central inflammation [55,115]. Among these metabolites, short-chain fatty acids (SCFAs) and bile acids (BAs) are particularly relevant. Experimental studies in mice have shown that SCFAs exert anorexigenic effects, with butyrate significantly reducing food intake by rapidly activating vagal afferent neurons [116,117,118].

A prospective observational study in RYGB patients revealed significant changes in circulating SCFA levels one year post-surgery, with a notable increase in propionate, butyrate, isobutyrate, and isovalerate, alongside a decrease in acetate, valerate, hexanoate, and heptanoate [119,120,121]. Additionally, animal studies have shown that gut bacteria post-BS (SG, RYGB, and BPD/DS) are associated with higher fecal SCFA levels, which positively correlate with peptide YY (PYY) secretion, a hormone that enhances satiety and appetite regulation [122]. Further experimental evidence suggests that SCFAs—particularly acetate, propionate, and butyrate—stimulate PYY release from intestinal L-cells, reinforcing satiety signals and appetite suppression [123].

Moreover, BS significantly modulates the gut microbiota, leading to an altered inflammatory profile at the gut level, with benefits extending to reduced neuroinflammation via the microbiota–gut–brain axis [55,124].

Additionally, bile acids (BAs), another group of metabolites produced by the gut microbiota, exhibit significant changes after bariatric surgery (BS). A meta-analysis of 289 patients by Zhang et al. reported higher fasting BA levels in RYGB patients, whereas SG patients did not display these differences. This discrepancy suggests that BA alterations may be linked to the formation of a biliary circuit and distinct intestinal microbiota changes specific to RYGB versus SG [125]. The Takeda G protein-coupled receptor 5 (TGR5) has been identified as a key regulator of the postprandial GLP-1 response. Activation of TGR5 by BAs stimulates GLP-1 secretion, highlighting a specific signaling mechanism mediating this effect [126]. An experimental study in animal models demonstrated that BA-induced GLP-1 release in intestinal cells enhances insulin secretion and satiety, thereby improving glucose regulation and reducing food intake [127]. However, while gut peptide levels (e.g., GLP-1, PYY) rise rapidly post-BS, plasma BA concentrations increase more gradually, with significant elevations observed only after one year. This suggests that BAs may not be the primary mediators of the early post-surgical surge in gut peptides [128,129].

**Figure 2:**
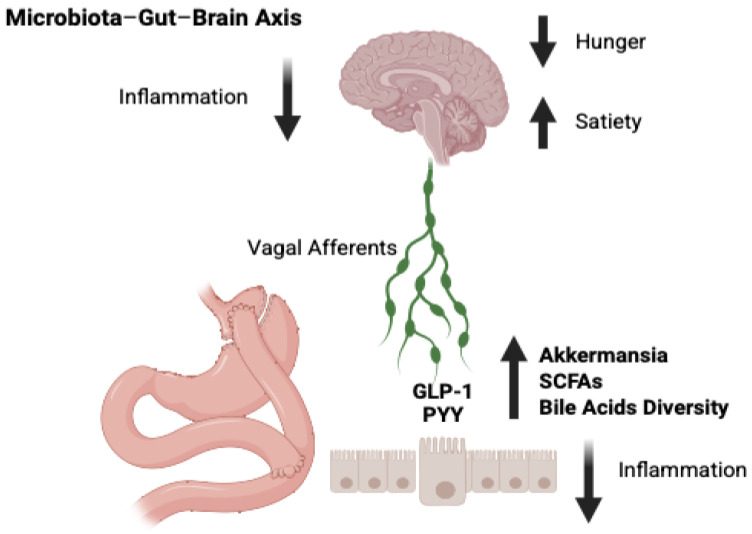
Changes in the microbiota after BS, with increased Akkermansia, decrease inflammation at peripheral and cerebral levels, influence the diversity of bile acids and metabolism of SCFAs, which exert their effect on enteroendocrine L-cell receptors with increased release of GLP-1 and PYY, which through vagal afferents alter neuronal activity modulating appetite and satiety. SCFAs: short-chain fatty acids; GLP-1: glucagon-like peptide-1; PYY: peptide tyrosine-tyrosine; ↑ increased; ↓ decreased [30,116,132,133].

Shifting focus to appetite and food preferences, one study identified three significant correlations between microbial composition and eating behaviors: Hunger was positively correlated with Enterococcus and Odoribacter; the desire for sweet foods was negatively correlated with Akkermansia [130,131]. Additionally, a systematic review reported that taste sensitivity to sweet and fatty stimuli increases post-BS, accompanied by a decreased hedonic (pleasure) response to these flavors. Moreover, enhanced olfactory acuity was observed, which may influence food preferences and consumption patterns, ultimately aiding in weight control [38]. All these gut microbiota alterations post-BS are summarized in Figure 2.

### 6.3. Mechanisms of Microbiota-Induced Changes in Neurohormonal Signals

RYGB and SG surgeries facilitate the arrival of bile acids (BAs) to the distal small and large intestine, where they stimulate enteroendocrine cells that enhance the secretion of GLP-1 and PYY [134]. Additionally, the direct delivery of nutrients to the distal intestine after BS leads to a further increase in GLP-1 and PYY secretion by L-cells [64]. A randomized prospective trial demonstrated that patients undergoing RYGB and SG experienced a significant rise in intestinal peptides (GLP-1 and PYY) within one week and three months post-surgery, indicating a rapid hormonal response following these procedures [128]. While GLP-1 secretion is enhanced after both SG and RYGB, RYGB generally produces higher GLP-1 levels. However, a randomized prospective study found that this difference was not statistically significant at three months post-surgery [135]. A meta-analysis evaluating SG patients reported improved GLP-1 secretion, which contributed to enhanced satiety and insulin sensitivity. Similarly, PYY levels played a key role in appetite regulation and satiety. The study, with a mean follow-up period of approximately 11.7 months, provided insights into long-term hormonal adaptations post-BS [136].

Similarly, an experimental study in mice found that bile diversion to the ileum led to significant alterations in gut microbiota, notably an increase in Akkermansia muciniphila. This bacterium is associated with enhanced gut health and is inversely correlated with intestinal inflammation, suggesting a potential role in metabolic regulation. These effects appear to be mediated through activation of the farnesoid X receptor (FXR) and stimulation of GLP-1 secretion [137].

A systematic review and meta-analysis analyzing fecal samples found that weight loss was significantly associated with an increase in α diversity of the intestinal microbiota (SMD: 0.4, 95% CI: 0.2–0.6, *p* < 0.0001) and a reduction in intestinal permeability (SMD: −0.7, 95% CI: −0.9 to −0.4, *p* < 0.0001). Additionally, there was a notable increase in the relative abundance of Akkermansia muciniphila [138]. A prospective non-randomized controlled study further identified significant post-RYGB increases in Streptococcaceae, Akkermansiaceae, and Veillonellaceae, which were positively correlated with GLP-1 levels. This suggests that gut microbiota may influence incretin hormone secretion via short-chain fatty acids (SCFAs) and bile acids (BAs), both of which stimulate GLP-1 release from intestinal L cells [139]. A similar effect was observed when Akkermansia muciniphila was introduced into mice [140]. Likewise, changes in gut microbiota post-BS in rodents were linked to higher fecal SCFA levels, which showed a positive correlation with PYY levels [122].

Bile acids (BAs), particularly deoxycholic acid, activate the TGR5 receptor, which is expressed in various tissues, including the hypothalamus, brown adipose tissue, and gut. The activation of hypothalamic TGR5 has been shown to reduce food intake, whereas its downregulation leads to increased eating, suggesting a direct role in appetite control [127]. Beyond the hypothalamus, TGR5 activation in the gut and peripheral nerves also contributes to appetite regulation, indicating that BAs influence appetite both directly via the hypothalamus and indirectly through the gut–brain axis [127]. The connection between gut microbiota, BAs, and GLP-1 production was further demonstrated in an experimental study in obese mice after SG by Chaudhari et al. This study showed that the microbial metabolite lithocholic acid (LCA), upon transport to the liver, activates the vitamin D receptor (VDR), converting BAs into their sulfurated form, 7-sulfate acid (CA7S), which in turn stimulates GLP-1 production [141]. Additionally, a meta-analysis involving 1916 participants found that changes in gut microbiota composition were linked to reduced intestinal permeability, which may mitigate the inflammatory response [138].

Inflammation at the level of the nodose ganglion may impair the responsiveness of vagal afferent neurons to gut-derived signals, including PYY, GLP-1, and CCK [123]. In an experimental study on high-fat diet-induced obese rats, RYGB surgery was found to reduce hypothalamic inflammation and microgliosis, which was associated with improved leptin sensitivity. These findings suggest that RYGB alters gut microbiota, leading to changes in circulating factors that influence hypothalamic inflammation, leptin signaling, and appetite control. This contributes to a better understanding of how RYGB sustains long-term appetite suppression. However, it is important to note that the study primarily focused on short-term outcomes [142,143].

### 6.4. Implications for Long-Term Dietary Habits

The mechanisms through which bariatric surgery (BS) induces weight loss extend beyond caloric restriction and malabsorption, with altered gut hormone signaling playing a crucial role [56]. Gut–brain communication is central to this process, as sensory information from the gut is transmitted via the vagus nerve, a key mediator of the changes in food preferences following RYGB. These changes impact both homeostatic (energy balance regulation) and hedonic (reward and pleasure-related) feeding circuits. Notably, vagal activity in the brain appears to be modulated after BS, influencing both the physiological need for food and the desire to eat [144]. Among the L-cell-derived gut hormones, GLP-1 and PYY are likely the primary contributors to post-surgical weight loss. GLP-1, in particular, is crucial for long-term appetite regulation and behavioral changes [56]. Interestingly, in a longitudinal observational study, even patients who experienced weight regain a decade after undergoing RYGB maintained elevated GLP-1 levels, suggesting a potential role in sustaining improved metabolic outcomes despite partial weight recurrence [145].

A systematic review by Ahmed et al. highlighted significant changes in taste perception following bariatric surgery (BS), including a decreased preference for sweet taste stimuli and an increase in olfactory acuity [38]. These findings suggest that post-surgical patients not only perceive tastes differently but also exhibit altered emotional responses to them, which could play a key role in long-term eating behavior and weight control. In a study by Søndergaard et al., which assessed the impact of RYGB and SG on food preferences and weight loss over 18 months, researchers found that overall food preferences remained unchanged. However, specific changes in food intake were associated with weight loss, indicating that individual variations in food preferences may serve as important predictors of postoperative weight outcomes. The small sample size of the study, however, limits the strength of these conclusions [146]. Additionally, a cross-sectional study conducted 24 months post-BS observed significant changes in food preferences. However, no strong associations were found between olfactory preferences and changes in body mass index (BMI). Interestingly, while changes in taste preferences were linked to lower BMI early after BS, these effects tended to revert to pre-surgical levels over time [147].

## 7. Effects of Bariatric Surgery on Taste Perception

### 7.1. Changes in Taste Sensitivity

The anatomical changes induced by bariatric surgery (BS) create a direct passage of food to the jejunum through the surgical bridge or an accelerated gastric transit due to the reduced stomach surface. These alterations trigger a cascade of mechanical, endocrine, and neurohormonal changes, impacting appetite, food preferences, and taste perception [83]. The complexity of taste perception makes it challenging to conduct objective and comparable evaluations, as it is influenced by all five senses—primarily taste and retronasal smell, which activate the olfactory epithelium [83]. As a result, individuals often struggle to distinguish whether the taste they perceive is due to olfactory activation or gustatory input, frequently confusing “flavor” with taste [83]. Additionally, taste perception consists of two key components: a discriminatory sensory component, which determines quality and intensity, and a hedonic component, which governs pleasure or repulsion toward specific flavors [83].

The use of surveys to evaluate changes in taste discrimination has yielded mixed results, with a general trend toward increased sweetness intensity; however, responses remain highly variable [83]. The Taste Change Survey has been widely used to indirectly assess taste perception alterations, yet findings have been highly inconsistent. Both increased and decreased sensitivity to sweet taste have been reported following sleeve gastrectomy (SG) and Roux-en-Y gastric bypass (RYGB) [83]. This variability may be attributed to several factors, which include the following: differences in surgical techniques, which do not directly affect peripheral taste innervation but likely influence taste perception through indirect mechanisms (to be discussed later); underreporting in survey responses, potentially compromising the reliability of results [83]; and other questionnaires that failed to detect significant changes in sweet taste perception [19,83,148]. Regarding fatty foods, an increase in taste intensity was reported within the first year post-surgery [83,88,148]. However, a decreased desire for sweet and fatty foods was observed, particularly in the first year post-surgery. This change was more pronounced in patients who successfully reached their weight-loss goals compared to those with suboptimal weight loss [83,148]. Additionally, changes in sweet and fatty taste perception were found to be more frequent in RYGB patients than in SG patients [83,88,148].

When analyzing taste perception thresholds, results remained inconsistent—some studies reported increased sensitivity to sweetness, while others found no significant changes. The subjectivity of individual responses and variability over time made it difficult to establish definitive conclusions [83,148,149]. 

### 7.2. Taste Preferences and Food Choices

Inconsistencies among studies on taste perception have not been shown to have a significant impact on weight loss or eating behavior changes [150]. Instead, weight loss is primarily linked to a reduction in total caloric intake [150]. Several factors influence changes in eating patterns, including study design and methodology; type of bariatric surgery performed; and physiological factors, such as the menstrual cycle [88,148]. Although there is a general trend toward lower consumption of sweet, fatty foods, and fruits, this effect has not been conclusively demonstrated [88,148,150]. This phenomenon aligns with the theory that hedonic perception of food, particularly sweets and high-fat foods, diminishes the desire and/or intake of these foods over time [88,148,151]. The underlying mechanism is linked to postprandial alterations in the secretion of key gut hormones—Ghrelin, Glucagon-like peptide-1 (GLP-1), and Peptide YY (PYY). These hormonal changes enhance satiety, and the brain interprets this response as a reward mechanism, reinforcing reduced intake of high-calorie foods [88,148]. Table 3 resumes the changes in taste perception.

### 7.3. Neural Mechanisms of Taste Modulation

Taste receptors are located on the tongue and palate and are innervated by the facial, glossopharyngeal, and vagal nerves [88]. The signals travel through these nerves to the nucleus of the solitary tract (NST), then to the thalamus, and finally to the primary taste cortex, where different taste modalities are processed and coded. These processes can be categorized into three distinct domains (Table 3) [88]: 1. Sensory domain—involves taste perception and identification; 2. Hedonic domain—governs the pleasure or aversion associated with taste; 3. Physiological domain—regulates appetite and metabolic responses to taste stimuli.

Taste rewards convey information related to food appeal and help guide appropriate eating behavior. The orbitofrontal cortex plays a crucial role in encoding the reward value of food stimuli [152]. Food reward is divided into two components: 1. Appetitive (“wanting”)—the motivation to seek and consume food; 2. Consummatory (“liking”)—the pleasurable response to eating [88]. Studies using MRI have shown reduced activation in the mesolimbic brain reward areas and the prefrontal cortex, which are responsible for motivation in response to sensory stimuli [152]. According to Smith et al., this reduction has been linked to caloric restriction, supporting the theory that changes in eating patterns occur post-surgery [88,152].

However, follow-up studies suggest that this effect may be transient, potentially serving as a prognostic factor for relapse [152]. Additionally, PET studies have shown a decrease in D2 dopamine receptors and alterations in dopamine secretion in the dorsal striatum [152]. Nevertheless, other similar studies have reported conflicting results [88,152,153].

## 8. Long-Term Implications for Weight Maintenance and Metabolic Health

Weight gain is a common complication after bariatric surgery (BS) and has been linked to multiple factors, including mental health conditions, surgical type and complications, poor dietary adherence, and reduced physical activity [97,154]. The incidence of weight regain (WR) varies depending on the definition used, the measurement parameters, and the time elapsed since surgery [97]. Studies report a median WR of 26.8% of maximum weight loss at five years post-surgery. Similarly, a median WR of 9.7% of presurgical weight has been observed at the same time point [97]. In other cases, WR ranges from 21 to 38% within 1–2 years post-procedure [155]. Additionally, weight regain of 8–13% post-surgery, followed by a weight loss plateau at 8–10 years, has also been reported [155]. The extent of weight loss varies by surgical type, with studies showing a mean weight loss of 6% to 22% at five years, depending on the procedure performed [156].

However, structural and neurohumoral changes are also observed in the central nervous system after bariatric surgery. Beyond metabolic and weight-related outcomes, bariatric surgery (BS) induces structural and neurohumoral changes in the central nervous system (CNS). A cohort study utilizing MRI to analyze the postoperative changes in bariatric patients reported a general increase in white matter (WM) density, particularly in the corona radiata, corpus callosum, cerebellum, brainstem, cerebellar peduncle, and cingulum [157]. At four months post-sleeve gastrectomy (SG), there was a notable increase in grey matter (GM) density compared to baseline, specifically in the right fusiform gyrus, right parahippocampal gyrus, right lingual gyrus, right amygdala, bilateral occipital and temporal cortices, postcentral gyrus, cerebellum, hippocampus, and insula [157]. These GM and WM improvements became more pronounced and widespread after one year, correlating strongly with metabolic improvements and postoperative weight loss [157]. Similarly, a cohort study by Custers et al., involving 133 post-BS patients, found that 52 participants exhibited at least a 20% improvement in global cognition within 24 months post-surgery. Additionally, the study observed increased physical activity, fewer depressive symptoms, and a reduction in antihypertensive medication use among patients [158]. While brain structure and perfusion generally decreased in most regions after BS, hippocampal and white matter volumes remained stable. Moreover, the temporal cortex exhibited greater thickness, further highlighting the potential neurocognitive benefits of BS [158].

Regarding the neurohumoral changes after bariatric surgery, several mechanisms have been proposed to explain them. Studies in animal models have shown alterations in neuropeptide Y (NPY) expression, including the upregulation of AGRP/NPY in the arcuate nucleus (ARC) [42]. Additionally, changes in postprandial cholecystokinin (CCK) levels have been reported in BS patients, potentially influencing NPY regulation [42]. BS also appears to affect orexin levels, both in the early postoperative period and before significant weight loss occurs. Nonetheless, these changes can occur in different directions. Some patients experience a decline in orexin levels, while others exhibit an increase following biliopancreatic diversion with duodenal switch (BPD/DS), a phenomenon associated with improved lipid and glycemic profiles [159]. Moreover, a study by Cigdem et al. found a substantial drop in weight accompanied by a decrease in orexin levels following laparoscopic gastric bypass (GB) [160].

According to what has been studied so far, these anatomical or neurohumoral changes have not been related to WR after BS. It would be interesting to carry out studies aimed at analyzing this association.

## 9. Discussion

This review provides a comprehensive analysis of the long-term physiological changes following bariatric surgery (BS), with a particular focus on neurobiological adaptations [69,161,162], gut microbiota alterations [60,163], and their collective impact on metabolic health [23]. A key strength of this work lies in its integration of findings from multiple disciplines, including neuroimaging [70,71], microbiome sequencing [163,164], and endocrinology, offering a holistic perspective on post-surgical metabolic regulation. Notably, this review is among the first to systematically explore the interplay between neural mechanisms, appetite regulation, and gut microbiota composition in the context of long-term post-bariatric outcomes. By synthesizing diverse methodologies and perspectives, this work provides valuable insights into the complex neuro-metabolic adaptations that occur following BS, highlighting key areas for future research and potential therapeutic interventions.

However, certain limitations should be acknowledged. As a narrative review, this work is inherently susceptible to selection bias and does not provide a systematic or meta-analytical assessment of the existing literature. Additionally, while the included studies offer valuable insights, they exhibit heterogeneous methodologies, varying follow-up durations, and limited sample sizes, making it challenging to draw definitive conclusions. Future research should prioritize large-scale, longitudinal studies that systematically evaluate postoperative changes over extended periods, ensuring greater methodological consistency and generalizability of findings.

Future research should focus on identifying predictive biomarkers to facilitate personalized post-surgical interventions. Additionally, long-term investigations into the effects of different bariatric procedures on gut–brain communication and metabolic homeostasis will be crucial for optimizing treatment strategies. Another essential area of exploration is the development of targeted probiotic or dietary interventions aimed at modulating gut microbiota composition to enhance metabolic outcomes and promote sustained weight loss.

By addressing these research gaps, future studies will contribute to a more personalized approach to post-bariatric surgery care, ultimately improving long-term health outcomes and minimizing complications.

## 10. Conclusions

Obesity is a highly prevalent condition linked to multiple comorbidities, including cardiovascular disease, diabetes, and an elevated risk of cancer. Bariatric surgery (BS) is a widely utilized therapeutic intervention that induces significant short- and long-term physiological changes.

Beyond its well-established effects on nutrient absorption and gastric volume reduction, bariatric surgery (BS) significantly alters the secretion of hormones and peptides involved in hunger and satiety, thereby influencing dietary patterns, eating behaviors, and taste preferences. Additionally, changes in gut microbiota composition and its interactions with the gut–brain axis may impact nutrient absorption and bile acid metabolism. Structural modifications in the central nervous system have also been reported, further highlighting the systemic effects of BS.

A thorough understanding of the physiological changes and their timelines following bariatric surgery (BS) is crucial for the multidisciplinary team managing patient care. Optimizing post-operative outcomes requires an integrated approach that includes personalized nutritional strategies, continuous gut microbiota monitoring, and long-term neurocognitive assessments. Since BS affects appetite regulation, taste perception, gut microbial composition, and overall metabolic responses, interventions should incorporate targeted nutritional supplementation, multi-strain probiotics, and tailored dietary education programs. Additionally, long-term management should include sustained endocrine and metabolic monitoring, alongside behavioral interventions to enhance dietary adherence and prevent weight regain. Future research should prioritize the identification of reliable biomarkers to further personalize post-surgical care, ensuring improved metabolic outcomes and minimizing long-term complications.

## Figures and Tables

**Table 1:** Brain effects produced by RYGB and LSG.

Brain Region	RYGB Effects	LSG Effects	Comparison
Dorsolateral prefrontal cortex (dlPFC) [67,68,69,71]	↑↑ activation	↑↑ activation, though less pronounced than RYGB.	RYGB results in better gains in cognitive control.
Nucleus Accumbens (NAc) and Striatum [67,68,69,71]	↓↓ Food cue reactivity, indicating lower reward sensitivity to food.	Variable effects, prevailing ↓↓ activity but less consistency.	RYGB has a greater influence on ↓↓ food-related reward processing.
Hypothalamus and Thalamus [67,68,69,71]	↑↑ Functional connectivity is linked to ↑↑ hunger regulation.	A moderate ↑ in connectivity	RYGB exhibits better normalization of hunger-related signals.
Para hippocampal/Fusiform Gyrus [67,68,69,71]	↓↓ activity in response to high-energy meal cues, indicating less attention to calorie-dense foods.	↓↓ activation	RYGB causes a greater ↓↓ of high-energy food cue processing.

RYGB: Roux-en-Y gastric bypass; LSG: Laparoscopic sleeve gastrectomy; ↑↑ strong increments; ↑ increment; ↓↓ strong decrease.

**Table 2:** Changes in gut microbiota composition following bariatric surgery [105].

Before Bariatric Surgery [101,102]	After Bariatric Surgery [105,106,107,108]
↓ Bacteroidetes	↑ Bacteroidetes
↑ Firmicutes	↓ Firmicutes
	↑ Proteobacteria
	↑ Verrucomicrobia (*Akkermansia*)

↑ increased; ↓ decreased.

**Table 3:** Changes in taste perception following bariatric surgery.

Taste Domain	Objectives	Mechanism	Effect of BS
Sensory [88,152,153]	Transmits taste stimuli to the thalamus and primary taste cortex, responsible for identifying and discriminating flavors.	Nerve fibers of the tongue transmit taste stimuli to the thalamus and the primary taste cortex.	No effect
Hedonic [88,152,153]	Integrates sensory input and modulates desire, reward, or aversion to flavors through cortical and mesolimbic system feedback.	Processes sensory input (stimuli from the tongue, vision, and olfactory nerves in the secondary taste cortex, generating feedback with the primary cortex and the mesolimbic system) and modulates reward pathways via dopamine secretion, reducing the drive for sweet or fatty foods.	↓ dopamine secretion
Physiological [88,152,153]	Regulates digestive processes, such as salivation and hormone secretion	Neuroendocrine stimulation.	↑ GLP-1 and PYY which participate in dopamine secretion

BS: Bariatric surgery; GLP-1: Glucagon-like peptide-1; PYY: Peptide tyrosine-tyrosine; ↓ decreased, ↑ increased.

## Abbreviations

The following abbreviations are used in this manuscript:

BAs	bile acids
BMI	body mass index
BS	bariatric surgery
BPD/DS	bilio-pancreatic diversion with duodenal switch
BPD	bilio-pancreatic diversion without duodenal switch
CDC	Center of Disease Control
DEBQ	Dutch Eating Behavior Questionnaire
FXR	farnesoid X receptor
GB	gastric banding
GI	gastrointestinal
GM	grey matter
GLP-1	glucagon-like peptide-1
LCA	lithocholic acid
LSG	laparoscopic sleeve gastrectomy
NST	nucleus of the solitary tract
NPY	neuropeptide Y
PYY	peptide tyrosine-tyrosine
RYGB	Roux-en-Y gastric bypass
SMD	Standard Media Deviation
SCFAs	short-chain fatty acids
SG	sleeve gastrectomy
TFEQ	Three-Factor Eating Questionnaire
TGR5	takeda G protein-coupled receptor 5
WL	weight loss
WM	white matter
WR	weight regain
